# Transfer Across Episodes of Analogical Reasoning: The Role of Visuo-Spatial Schemas

**DOI:** 10.5334/joc.408

**Published:** 2025-01-06

**Authors:** Jairo A. Navarrete-Ulloa, Maximo Trench

**Affiliations:** 1 Education Sciences Institute, Universidad de O’Higgins, Rancagua, CL; 2 Psychology, Universidad Nacional del Comahue University Regional Centre Bariloche, San Carlos de Bariloche, AR

**Keywords:** analogy, meta-analogy, mapping, transfer, visuo-spatial schemas

## Abstract

The standard explanation of meta-analogical transfer posits that the predicate mappings generated during a first analogy episode get reused during subsequent instances of analogical reasoning. As this account fails to predict the empirical result that only mappings between similar concepts get reliably transferred, other psychological mechanisms seem to be at play. Across three experiments, we obtained evidence suggesting that the carry-over of visuo-spatial schemas can also be involved in meta-analogical transfer. In Experiments 1 and 2, participants rated solutions to an ambiguous letter-string analogy whose alternative solutions involved different visuospatial operations. Prior to that, participants rated solutions to letter-string analogies aimed to elicit visuospatial operations that were either consistent, inconsistent or unrelated to the visuospatial operations underlying the later problem. Participants granted higher scores to solutions whose underlying visuospatial operations matched those elicited by the preparatory analogies. In Experiment 3, participants rated solutions to the target ambiguous analogies after watching short animations representing the visuospatial representations presumed to have been elicited by the preparatory analogies of Experiments 1 and 2. The fact that these animations biased participants’ ratings in the same manner as in the previous experiments provides further evidence that dynamic visuo-spatial schemas can play a role in meta-analogical transfer.

Analogical reasoning plays a central role in activities as diverse as prediction (Hofstadter & Sander, 2013), persuasion (Blanchette & Dunbar, 2001), problem solving (Kurtz & Loewenstein, 2007), creative invention (Christensen & Schunn, 2007), scientific discovery (Gentner et al. 1997) or instruction (Richland et al., 2007; Navarrete-Ulloa & Muñoz-Rubke, 2022). Upon establishing a set of correspondences between elements that play parallel roles in two situations, unmapped elements from the better understood situation (base analog) can be inductively projected onto the least understood situation (target analog), thus enhancing its representation. Given its transversality, several authors have claimed a central role for analogy in intelligence (Correa-Beltran et al, 2016; Lovett et al. 2010) and in cognition at large (Hofstadter, 2001; Gust et al., 2008), spanning from primates (e.g., Oden et al., 2001) to humans (Gentner, 2003) to artificial intelligence (Hofstadter & FARG, 1996; Kolodner, 1992).

Despite philosophers’ initial skepticism about the prospects of unveiling the mechanisms that subserve analogical thinking (see e.g., Fodor, 1983), subsequent theoretical accounts of the analogical subprocesses of retrieval, mapping, and inference have been successfully modeled using either purely symbolic (e.g., SME, Falkenhainer et al., 1989; MAC/FAC, Forbus et al., 1995, Navarrete et al., 2017) or symbolic-connectionist architectures (e.g., ACME, Holyoak & Thagard, 1989; LISA, Hummel & Holyoak, 1997; 2003). Behavioral experiments have been crucial in unveiling the cognitive mechanisms that give rise to analogical reasoning, thus informing the development of artificial information-processing systems.

The present research aims to delve into the cognitive mechanisms that subserve transfer across instances of analogical reasoning. Even though analogy represents the default strategy by which knowledge can be flexibly transferred across similar problems, proportional analogies are noteworthy in that solutions to a wide variety of analogy problems ultimately rely on a single heuristic: finding which element of a target situation plays the role of a specific component in a source situation. While solving proportional analogies such as “*charcoal* is to *furnace as wood* is to….” requires a wealth of word knowledge, analogies like “*abc* is to *abd* as *ijk* is to…” are comparatively less dependent on this kind of knowledge, and more dependent on fluid intelligence and executive functions. Sequences of proportional analogies, therefore, figure prominently as valid and reliable tests of crystallized and fluid intellectual capacities, and their alleged reliability is based on the implicit assumption that solutions to a proportional analogy could hardly influence solutions to subsequent episodes of analogical reasoning.

Counter to this widespread assumption, a growing number of recent studies have documented that exposure to proportional analogies can affect subsequent performance on other types of analogy problems. Vendetti et al. (2014) had two groups of participants solve a series of either near (charcoal:furnace::wood:?) or distant (charcoal:furnace:food:?) analogies prior to receiving a series of visual analogies in which the same relation held among the characters of two scenes (e.g., a cat chasing a mouse and a dog chasing a cat, plus other distracting elements). After one character in the first scene (e.g., the cat) was visually highlighted, researchers asked for the character in the second scene that matched the highlighted agent in the first scene. Results showed that participants who had solved distant proportional analogies during the first phase of the experiment were more likely to select the relational match (i.e., dog) over the perceptual match (i.e., cat) than those in the near condition. The authors interpreted this effect as demonstrating that the activity of solving distant proportional analogies elicited a mindset to look for relational features of subsequent stimuli. In a recent study, Simms and Richland (2019) had 4-year-old children complete an orienting task consisting of either actively generating solutions or passively listening to solutions for visually presented proportional analogies. Afterward, children were presented with a series of visual scene analogies similar to those used by Vendetti et al. (2014). Participants who had engaged in actively seeking solutions to the proportional analogies were more likely to prefer the relational choice in the subsequent scene analogies task than those in the passive condition, and roughly as likely as 4-year-old participants of a prior study (i.e., Richland et al., 2006), who were explicitly instructed to select the relational matches. Taken collectively, the analogical mindset studies reviewed above represent strong evidence that engaging in analogical tasks promotes a mindset that facilitates solutions to subsequent instances of analogical reasoning by highlighting the relational features of the targets.

## The Transfer of Predicates Account of Meta-Analogical Transfer

Having by now been well-established that general aspects of analogical processing (e.g., a focus on relations rather than objects) can be carried over across instances of analogical reasoning, the question arises as to whether more specific aspects of the reasoning that underlies a particular analogical comparison can also be transferred to subsequent analogies. As an example, consider Hoftstadter’s (1996) analogy between Nancy Reagan, who by 1980 was the first lady of the United States, and somebody playing a similar role in England. Amongst potential candidates like Queen Elizabeth or Margaret Thatcher, the prime minister’s husband Dennis Thatcher seemed more analogous by drawing a similar relation to political power. Once the concept “man” associated with Dennis Thatcher is placed in correspondence with the concept “woman,” associated with Nancy Reagan, one theoretical question concerns whether this association gets retained for reuse during subsequent analogies. If this woman-to-man mapping gets reactivated while solving subsequent analogical problems such as “who is the Albert Einstein of France?”, then it could predictably facilitate answers such as “Marie Curie”.

In an early behavioral study of transfer between episodes of analogical reasoning (i.e., *meta-analogical transfer*), Burns (1996) confronted participants with the letter-string analogy “If *abc* were changed to *abd*, how would you change *kji*^1^ in a similar way?”. He observed that responses produced by participants who solved it in the first place differed from those from participants who solved it after being exposed to the *abc:abd::mrrjjj* analogy. When the *abc:abd::kji* analogy was presented first, participants granted similar goodness scores to solutions *kjj* (based on the successor-to-successor mapping), *lji* (based on the left-to-right and the sequence-to-sequence mapping), and *kjh* (based on the successor-to-predecessor mapping). In contrast, participants who confronted the above problem after being exposed to the *abc:abd::mrrjjj* analogy overwhelmingly favored the *kjj* solution. As defensible solutions to the *abc:abd::mrrjjj* analogy depend on the successor-to-successor mapping, the increased number of participants selecting the *kjj* solution to the *abc:abd::kji* analogy after having confronted the previous analogy suggested that participants carried over the successor-to-successor mapping from the first analogical episode to the second. To assess the generality of this finding, a subsequent experiment set forth to determine whether transfer between episodes of analogical reasoning can also occur when the mapping involved in the preparatory analogy connected two different predicates, as *man*↔*woman* in the Thatcher analogy. In this experiment, a successor-to-predecessor mapping was enforced by way of asking participants to justify the *kjh* answer to the *abc:abd::kji* analogy. Afterward, participants faced the analogy *abc:abd::xyz*, and rated the goodness of the alternative answers *wyz* and *xyd*. The fact that participants in the above condition favored solution *wyz* was taken as evidence that the successor-to-predecessor mapping was transferred from the first analogical episode to the second. In view of these results, Burns concluded that the predicate mappings elicited during a first analogy get retained in long-term memory for further reuse during subsequent instances of analogical reasoning. Even though computational models of analogical mapping (e.g., *Copycat*, Hofstadter & Mitchell, 1994; LISA, Hummel & Holyoak, 2003; SME, Falkenhainer et al., 1989) have not yet incorporated a means for storing past mappings in long-term memory, their proponents have manifested that said addition would represent the most natural way to go if one wants computer models of analogical thinking to keep up with human ability to learn from previous performance (Keith Holyoak, personal communication; Hofstadter, 1996).

Despite the intuitive appeal of the “transfer of predicate mappings” hypothesis, later studies on meta-analogical transfer yielded inconsistent results. In another study using letter-string analogies, Pulido (2002) replicated the effect of meta-analogical transfer between problems admitting a successor-to-successor mapping, and generalized it to pairs of problems involving predecessor-to predecessor mappings. However, no effects of meta-analogical transfer were observed between problems involving predecessor-to-successor mappings. Taken collectively, the results of Burns (1996) and Pulido (2002) suggest that while mappings between similar concepts are transferred across instances of analogical reasoning, transfer across problems involving mappings between dissimilar concepts is less reliable (see Pulido et al., 2010 for a review). If the carry-over of predicate mappings were sufficient to account for meta-analogical transfer, the transfer effects should be observed not only among analogy episodes whose mappings involve similar predicates, but also among analogy problems whose mappings involve dissimilar predicates, as in Hofstadter’s meta-analogy. In light of the evidence that only mappings between similar concepts are reliably transferred across instances of analogical reasoning, it seems likely that other mechanisms different from the carry-over of predicate mappings may be at work when solving sequences of letter-string analogies.

A first objective of the present experiments was to enlarge the still narrow set of studies showing that transfer between episodes of analogical reasoning involves not only a general mindset to attend to relations, but also the carry-over of problem-specific features across subsequent episodes of analogical problem-solving. By assessing the operation of cognitive mechanisms different from the carry-over of predicate mappings, the present research also aims to provide a more comprehensive account of the results obtained in the literature on meta-analogical transfer.

## A Visuospatial Priming Account of Meta-Analogical Transfer

In the years that passed since the transfer of predicate mappings hypothesis was put forward, a handful of studies have started to address the effects of visuo-spatial animations on analogical problem-solving (Grant & Spivey, 2003; Goldstone et al., 2010; Kubricht et al., 2017; Pedone et al., 2001; Thomas & Lleras, 2007), and in problem-solving at large. Pedone et al. (2001) obtained that a dynamic animation involving convergent arrows helped participants propose convergent solutions to Duncker’s (1945) radiation problem, where an available ray can destroy a patient’s tumor, but with the drawback that rays of the required intensity would also harm the surrounding tissues. In a related study, Grant and Spivey (2003) obtained similar facilitation effects by inducing a converging pattern of attention and eye movements over a diagram of the radiation problem. In turn, Thomas and Lleras (2007) confirmed that the factor that conferred said advantage was direction of the saccades and not merely attention to an area of interest (the surrounding tissues), and also reported that for the great majority of participants this facilitation occurred without conscious awareness.

The effect of visuospatial and visuomotor schemas on complex cognition was also observed outside the domain of insight problem-solving. In a study about how participants solve algebraic equations, the equations to be solved were superimposed on a moving background grating whose direction was either consistent or inconsistent with the “spatial transposition strategy” that participants needed to perform (e.g., moving the number 8 from the left to the right of the equality in 4*y+8 = 24). Goldstone et al. (2010) documented that both the speed and accuracy of performance were affected by whether the direction of the concurrent grating was consistent vs. inconsistent with the intended transposition, a result that was interpreted by the authors as demonstrating the embodied basis of a type of processing largely regarded as independent from perception (see also Navarrete et al., 2018 for examples of how numbers’ spatial alignment influences their learning).

In a related study using an abstract “bouncing-balls” display to prime either a vertical or a horizontal dimension, Livins et al. (2016) demonstrated that this seemingly unrelated display facilitated solving scene analogies whose central relation was consistent with the primed dimension (e.g., a horizontal bouncing facilitated solving cross-mapping analogies involving “chasing”). In another experiment of the same study, these dynamic vertical vs. horizontal visuospatial displays primed participants’ interpretation of ambiguous arrays of abstract figures. For example, an ambiguous array wherein an occluding circle is positioned above and to the right of an occluded square was more frequently categorized as involving a “right of x” disposition after a horizontal than a vertical “bouncing balls” animation. Taken collectively, the above studies provide evidence that dynamic visuospatial schemas can inadvertently alter responses to target problems ranging from the conceptual to the perceptual and from the concrete to the very abstract.

Based on the wide range of high-level activities that are subject to visuospatial priming, in this paper we gather further evidence to examine whether the activation and carry-over of dynamic visuo-spatial schemas provides a more comprehensive account of the observed patterns of transfer between letter-string analogy problems. For example, consider the observed transfer from the *abc:abd::mrrjjj* analogy to the *def:deg::ijk* analogy. When processing the first analogy, the *abc:abd* transformation could perhaps be conceptualized by participants by simple visual operations akin to how a mechanical odometer would replace a “7” by an “8” by rotating a numbered wheel, or to how attention would shift from a letter to its right-side neighbor in an image of the learned standard letter sequence. Once visual operations of this kind are reused to change the rightmost element (or chunk of similar elements) in the same way as was used to convert *abc* to *abd*, they might remain available for further analogies therefore favoring solutions that involve the application of said visual transformation to the target item. Unlike this kind of letter-string problem, where the same elemental operation applies uniformly to the base and the target, analogies that require a mapping between dissimilar predicates cannot be easily resolved via this elemental visuo-spatial heuristic. Therefore, transfer from one analogical episode to a subsequent one becomes less likely, eventually giving rise to the inconsistency of results that can be observed in the literature. The present experiments were designed to evaluate the hypothesis that meta-analogical transfer can be explained by the activation and carry-over of dynamic visuo-spatial schemas. To assess the effects of perceptual-like structures on meta-analogical transfer, we will examine transformations that go beyond the exclusive deployment of the “successor” and “predecessor” transformations that characterize the existing literature. Therefore, the present study also contributes to the existing literature by broadening the spectrum of letter-string problems available for future research.

By using a rating task akin to the one employed by Burns (1996) and Pulido (2002), in the first two experiments of the present study we began by confronting participants with a set of letter-string analogies (Experiment 1) or a single letter-string analogy (Experiment 2) which, according to condition, involved either a simple rotation about the vertical axis (henceforth, *mirror rotation*) or a comparatively less intuitive “left shift” operation (henceforth, *shift operation*). After providing goodness scores to the correct solution plus distracters, participants had to rate solutions to ambiguous letter-string analogies that could be successfully solved by either the visuo-spatial operation elicited by the previous analogy, or by the alternative visuospatial schema. In both experiments, we predicted strong meta-analogical transfer effects of a kind that would be difficult to explain parsimoniously in terms of the carry-over of predicate mappings. In Experiment 3, participants were exposed either to the shift or to the mirror operation through a brief computer animation whose elements were perceptually and contextually unrelated to letter-string analogies. After this, all participants faced the ambiguous letter-string analogy and rated solutions that were either consistent or inconsistent with the previously observed animation. We predicted that exposure to the left shift animation would increase goodness scores to the left shift solution as compared to the other animations, even among participants who failed to spontaneously notice a connection between the visual animation and the analogy problem presented afterward. Taken collectively, the results of the present experiments add to a still narrow set of studies showing that transfer across episodes of analogical problem-solving involves not only a general mindset to focus on relational structures, but also the carry-over of problem-specific aspects of the analogies themselves. More crucially, the cognitive mechanism here proposed helps explain some empirical discrepancies in the literature, thus yielding a more comprehensive account of meta-analogical transfer.

## Experiment 1

### Method

#### Participants and Design

An initial sample of 114 undergraduate students at a public university participated for course credits. They were randomly assigned to the shift priming condition (*N* = 40), the mirror priming condition (*N* = 38) and the no-priming condition (*N* = 36).

#### Materials and Procedure

The experiment was administered through the Qualtrics online platform. Participants worked individually at the computer laboratory of the University, and completed the subsequent tasks at their own pace. The introductory phase of the experiment had the dual objective of collecting demographic data and familiarizing participants with the interface for answering analogy tasks. After a demographics section, the screen presented participants with the four-word analogy “*Chile:Santiago :: Argentina:?,”* followed by four answer options. The four alternatives had preset values (Buenos Aires-80, Brasil-0, Mendoza-20, and Paris-0) and a brief text explaining why Buenos Aires should have the highest score (capital of Argentina), followed by Mendoza (city of Argentina).

The next section (priming phase) was intended to activate a specific visuospatial operation that could potentially favor perceptually-consistent solutions to ambiguous the letter-string analogies that were presented during the subsequent (testing) phase. In the priming phase of all conditions, the software presented participants with a sequence of three proportional analogies sharing a similar solution schema. In the “left shift” priming condition, the intended schema involved in the problems *abc:cab::123:, mno678:omn867::def234:*, and *pqr:rrppqq::xyz:* was a spatial operation that consisted in moving the rightmost object of a series to the leftmost position. After receiving each problem, participants had to assign goodness ratings to four possible solutions including a left-shift alternative (e.g., *312*) and three distractors. Participants had to express their preference for each alternative by adjusting a sliding bar ranging from 0 to 100, but with the constraint that the ratings of all four alternatives to a given problem should add up to 100. In the mirror priming condition, the schema underlying the problems *abc:cba::123:, mno678:onm876::def234:*, and *pqr:rrqqpp::xyz:* was a kind of rotation about the vertical axis wherein the leftmost and the rightmost elements (or groups of elements) in a series exchanged their positions. For each problem, participants had to assign goodness ratings to a mirror solution (e.g., *321*) plus three distractors. In the no-priming condition, participants received the problems *human:lungs::fish:, person:house::bird:* and *fish:water::student:*, whose solutions did not appeal to any specific visuo-spatial transformation, but instead rely on abstract relational concepts. As in the other conditions, participants had to assign goodness scores to one correct answer plus three distractors. The objective of the no-priming condition was to match the priming conditions in terms of engaging participants in reasoning about proportional analogies, but without eliciting any of the visuospatial operations intended to bias participants’ solutions to the problems to be presented during the testing phase. To maximize the odds of detecting a transfer effect, our assessment of meta-analogical transfer relied on comparing the shift against the mirror priming conditions in terms of participants’ appraisal of the presented solutions to the test problems. The objective of the no-priming condition was mostly to reveal participants’ unbiased appraisal of these solution alternatives.

In order to assess the effect of the priming phase on the processing of subsequent analogies, the two letter-string problems included in the test phase of the procedure could be solved either through a shift or a mirror operation. For the first analogy (*pq89pq:qp89qp::xyz89xyz:*), participants had to assign goodness scores to a shift alternative (*zxy89zxy*), a mirror-alternative (*zyx89zyx*), and two distractor-alternatives (*yxz89yxz* and *xyz89xyz*). Likewise, for the second analogy (*aab12aab:baa12baa::cde12cde:)* participants had to assign goodness scores to a shift-alternative (*ecd12ecd*), a mirror-alternative (*edc12edc*) and two distractor-alternatives (*dce12dce* and *cde12cde*). This second analogy aimed to assess whether the effect of the priming manipulation would transcend its application to a first analogical episode. Unlike the example analogy presented during the introductory phase of the experiment, solutions to priming and test analogies had preset values of zero. The order of solution alternatives was randomized across participants, and the software recorded the response times for potential use as a control variable.

### Results and Discussion

As allocating high preference scores to distractor alternatives signals a lack of attention to the target task, participants whose preference scores for distractor alternatives were above two standard deviations from the mean (*N* = 21) were withdrawn from the analysis. Table 1 summarizes participants’ preference scores as a function of priming condition and type of solution.

**Table 1:** Preference scores as a function of priming condition and type of solution, Experiment 1.


TASK AND PRIMING CONDITION	N	TIME	SHIFT-SCORES		MIRROR-SCORES		DISTRACTOR-SCORES	
		
			MEAN	SD	MEAN	SD	MEAN	SD

1^st^ ambiguous analogy								

no-priming	33	82	12.2	23.9	80.9	29.5	6.9	15.1

mirror priming	31	86	12.1	24.8	79.1	33.3	8.8	19.6

shift priming	29	97.6	29.7	37.1	64.5	39.7	5.8	16.7

2^nd^ ambiguous analogy								

no-priming	33	94.6	29.3	38.1	64.2	37.7	6.6	14.2

mirror priming	31	79.2	25	39,8	67.9	41.3	7.1	17.4

shift priming	29	84.7	52.9	35.7	40.9	37.2	6.2	16.1


In the no priming condition, participants mean preference scores were of 12.2 for the shift alternative, of 80.9 for the mirror alternative, and of 6.9 for the distractors, thus confirming that the shift alternative, although defensible, was not granted high preference scores by this particular population. As the restriction of having scores of all solution alternatives add up to 100 points rendered participants scores for the mirror solution largely dependent on the scores received by the shift alternative, we restricted the statistical analysis of the priming effects to the scores received by the shift alternative, which was not naturally favored by participants of the no priming condition.

An ANCOVA analysis with condition as between-subjects factor and Response Time as a covariate revealed an effect of condition on participants’ preference scores for the shift solution [*F*(2, 89) = 3.26, *p* = .04, *η^2^_p_* = .08]. A planned comparison confirmed that participants whose priming analogy was intended to elicit a shift operation granted higher preference scores to the shift solution than participants whose priming analogy was intended to elicit a mirror operation, *t*(48.41) = 2.15,*p* = .037, Cohen’s d = .57, 95% CI [0.04, 1.09]. This result demonstrates that the preference scores for a shift-solution were higher when a previous set of problems involved a matching operation, as opposed to when the previous set of problems involved an inconsistent visuo-spatial schema.

In regard to the second analogy of the test phase, an ANCOVA analysis of variance with condition as between-subjects factor and response time as covariate revealed that the preference for the shift-alternative differed across conditions, *F*(2,89) = 4.75, *p* = .01, *η^2^_p_* = .10. A planned comparison showed that the difference between the mirror-priming and the shift-priming groups was significant, *t*(57.9) = –2.87, *p* < .01, Cohen’s *d* = .75, 95% CI [0.2, 1.29]). The fact that a transfer effect was also obtained with the second test analogy suggests that the purported activation of visuospatial schemas does not decay abruptly after the priming episodes. However, the fact that the contrast between the consistent and inconsistent priming conditions was numerically larger for the second analogy than for the first is also compatible with the hypothesis that participants’ response to the first ambiguous analogy somehow reinforced the effect of the priming manipulation.

The results of Experiment 1 are consistent with those of Burns (1996) and Pulido (2002), where the nature of solutions to an analogical episode primed the appraisal of solutions to a subsequent instance of analogical reasoning. Even though the observed transfer effects could conceivably be explained in terms of the transfer of predicate mappings, a more parsimonious explanation would be that once a simple visuospatial schema capturing the A:B transformation of the first analogy was applied to its “C” term in order to derive the unknown term “D”, its activation helped to disambiguate the second analogy.

As compared to prior studies, one limitation of the procedure employed in Experiment 1 to induce a bias towards a particular solution concerns the need to confront participants with three instantiations of the visuospatial operation we intended to highlight. Besides replicating the main finding of Experiment 1 with a more powerful sample, Experiment 2 aimed at assessing whether the same effect could be elicited by the presentation of a single problem during the priming phase.

## Experiment 2

### Method

#### Participants and design

An initial sample of 231 undergraduate students at a public university participated in the study for course credits. They were randomly assigned to the “left shift” priming condition(*N* = 83), the “mirror” priming condition (*N* = 86) and the unrelated priming condition (*N* = 62). A minimum sample size 64 participants per condition was established via setting a G*Power analysis (Faul et al., 2007) to achieve a power of 0.8 in detecting an effect size of 0.5, that is, an effect size slightly smaller than the one obtained in Experiment 1. The increase in the number of participants from Experiment 1 to Experiment 2 meets current recommendations for replication studies (e.g., Simonsohn, 2015).

#### Materials and procedure

The experiment was administered through the Qualtrics online platform at the computer laboratory of the University. Participants worked individually, and the administration was self-paced and free of time constraints. The demographics questions and the familiarization example were identical to those of Experiment 1. The priming phase was also similar to that of Experiment 1, with the difference that in each condition participants had to rate the solutions to one analogy problem instead of three. While in the shift-priming condition participants received the unambiguous letter-string analogy *mno678:omn867::def234:* along with a shift solution (*fde423*) and two alternative distractors, in the mirror-priming condition participants received the unambiguous letter-string analogy *mno678: onm876::def234:* along with a mirror solution (*fed432*) plus two distractor alternatives. In the no-priming condition, the solution to the problem *human:lungs::fish:* did not appeal to any visuo-spatial transformation, but rather to an abstract relational concept. In all three conditions, participants had to assign goodness scores to each of the presented solutions, with the restriction that the sum of the ratings should add up to 100.

During the test phase, participants of all conditions received the ambiguous letter-string problem *aab12aab:baa12baa::cde12cde:* together with a shift-alternative (*ecd12ecd*), a mirror-alternative (*edc12edc*) and one distractor-alternative (*dec12dec*). Participants had to adjust sliding bars to allocate preference scores for each of the presented solutions. The order of solution alternatives was randomized across participants, and the software recorded the response times for potential use as a control variable.

### Results and discussion

As in Experiment 1, participants (*N* = 12) whose preference score for the distractor alternative of the ambiguous analogy was above two standard deviations from the mean were withdrawn from the analysis. Table 2 summarizes participants’ ratings as a function of priming condition and type of solution. In the no priming condition, participants preference scores were 27 for the shift alternative (*ecd12ecd*), 69.5 for the mirror alternative (*edc12edc*), and 3.5 for the distractor alternative (*dec12dec*).

**Table 2:** Preference scores as a function of priming condition and type of solution, Experiment 2.


PRIMING CONDITION	N	TIME	SHIFT-SCORES		MIRROR-SCORES		DISTRACTOR-SCORES	
		
			MEAN	SD	MEAN	SD	MEAN	SD

no-priming	55	66.7	27	35.9	69.5	36	3.5	10.9

mirror priming	85	69.2	28.4	30	68.5	30.1	3.1	8.9

shift priming	79	66.1	53.7	35.5	44.1	35.7	2.2	7.3


Although higher than in Experiment 1, participants unbiased appraisal of the shift solution to the ambiguous problem was still visibly lower than that of the mirror solution. An ANCOVA analysis of variance with condition as between-subjects factor and time on task as covariate revealed a significant effect of condition on the preference scores granted to the shift-alternative, *F*(2,215) = 15.6, *p* < .001, *η^2^_p_* = .13. A planned comparison showed that the preference scores granted to the shift solution were higher when the priming problem was intended to involve this operation than when it involved an inconsistent “mirror” operation, *t*(153.24) = –4.91, *p* < .001, Cohen’sd = .78, 95% CI [0.45, 1.1]. Experiment 2 thus replicates the pattern obtained in Experiment 1, but with a larger sample of participants and a single priming analogy instead of three. Results lend additional support to the hypothesis that meta-analogical transfer effects can be explained by the activation and carry-over of dynamic visuospatial schemas that capture the relation between the A and B terms of the analogies.

Albeit intricate, one way of stretching the “transfer of predicate mappings” account to accommodate the results of Experiment 1 and Experiment 2 would be to accept the idea that the A:B and the C:D transformations within the priming and the test analogies are naturally captured by compact schemas such our “shift” and “mirror” operations, but to maintain that what is actually transferred across analogy episodes are not the schemas themselves, but rather the mappings that associate the A:B transformations with the C:D transformations of the priming analogy—e.g., shift(mno, omn) ® shift(def, fde) and shift(678, 867) ® shift(234, 423). When later confronted with the ambiguous analogy *aab12aab:baa12baa::cde12cde:*, the shift solution *ecd12ecd* would be preferred by participants over the mirror solution *edc12edc* by virtue of eliciting the “shift” operations that are necessary for projecting the previous set of analogical mappings (the mirror operations underlying the edc12edc solution do not allow transferring such mapping). Besides its complexity, one problem with this interpretation is that it would predict not only that the shift-primed group would assign higher goodness scores to the shift solution than the control group —a prediction confirmed by our results—, but also that the mirror-primed group would assign higher goodness scores to the mirror solution than the control group. Our results did not confirm this second prediction. As opposed to the transfer of predicate mappings interpretation, the more parsimonious account based solely on the activation and carry-over of visuospatial schemas can potentially explain this asymmetry by positing that, since the mirror interpretation represents the more natural interpretation of the test analogy (compare scores of solutions given by the no-priming group and the mirror-priming group), its elicitation in the mirror-priming condition was somewhat redundant.

In order to determine whether similar response patterns to those observed in Experiments 1 and 2 can be obtained through a procedure for which the transfer of predicate mappings could not possibly operate, in Experiment 3 the visuospatial schemas presumed to operate in the previous experiment were presented directly, that is, without being framed within a preparatory analogy. If these visuospatial animations prove capable of biasing solutions to the test problem despite not taking part in an episode of analogical reasoning, then the accumulated evidence would represent a strong case for considering the transfer of dynamic visuospatial schemas as one of the prevailing mechanisms underlying meta-analogical transfer.

## Experiment 3

### Method

#### Participants and design

A sample of 266 undergraduate students at a public University were randomly assigned to one of three conditions: shift-priming (*N* = 89), mirror-priming (*N* = 87) and no priming (*N* = 90). A minimum sample size 64 participants per condition was established via setting a G*Power analysis to achieve a power of 0.8 in detecting an effect size of 0.5.

#### Materials and procedure

The experiment was administered through the Qualtrics online platform at the computer laboratory of the University. Participants worked individually, and the administration was self-paced and free of time constraints. To further familiarize subjects with both the computer interface and the task itself, participants received three four-term word analogies, and were asked to assign their own goodness scores to the presented options. The familiarization examples were similar to those of Experiments 1 and 2, but without the restriction that the scores to all alternative solutions should add up to 100. We reasoned that while the prior restriction to have all scores add up to 100 effectively unveiled relative preferences among solution alternatives, it did not allow participants to express their intrinsic degree of agreement with any particular solution.

The priming phase of the procedure aimed to activate a different abstract concept in each group of participants. All participants watched a fifteen-second animation intended to represent an abstract visuospatial operation: After displaying a reverse counter (3-2-1) to attract participants’ attention, the screen showed a horizontally-laden array of three figures (circle, square, and triangle) inside a large rectangular box. The objects soon displayed an animated movement that differed according to condition. In the shift-priming condition, the triangle right-exited the central rectangle, then described a counterclockwise trajectory around the rectangle, and finally reentered the central panel through the left side, thus occupying the leftmost position in the array. In the mirror-priming condition, the video showed a 3D rotation of 180° carried around the middle vertical axis, such that the circle and the triangle exchanged locations. This animation visually represents an abstract operation that takes a series of objects and swaps the objects located at the first and last places. In the no-priming condition, the rectangle containing the initial array experienced a moderate trembling that shook the rectangle but did not modify the ordering of the geometric figures. To enforce participants’ attention to the animation, participants were warned that they would be able to watch the animation only once, and that they should be able to describe it. After participants watched the animation, they were asked to describe how the spatial configuration of the figures changed over time.

During the testing phase of the procedure, participants of all conditions confronted the ambiguous letter-string analogy *aab23aab:baa23baa::ghi23ghi:*, followed by three solution options: (a) *igh23igh*, a defensible solution whose underlying operation matched the shift animation; (b) *ihg23ihg*, a defensible solution whose underlying operation matched the mirror animation, and (c) *gih23gih*, a non-defensible solution serving as distractor. Participants rated the goodness of each solution sliding a bar from 0 to 100. The order of solution alternatives was randomized across participants, and the software recorded the response times for potential use as a control variable.

The test phase included two additional measures: a *post-task awareness questionnaire* and a *hinted-transfer control*. As in other studies of transfer (e.g., Schunn & Dunbar, 1996; Thomas & Lleras, 2009; Trench & Minervino, 2015), the post-task awareness questionnaire aimed to determine whether the information to be transferred had consciously come to mind during the transfer activity (i.e., “did you think of the animation, even if just briefly, while reading the last problem and/or assigning preference scores?”). The hinted transfer control aimed to assess the maximal extent to which participants are able and/or willing to alter their otherwise natural scores upon an explicit indication to use the perceptual animation as a basis for their goodness scores. To that end, the ambiguous analogy problem and its candidate answers reappeared on a subsequent screen, and participants were asked to reassign goodness scores to each solution based on the information conveyed by the animation.

### Results and discussion

Data from 9 participants whose preferences for distractor alternatives were above two standard deviations from the mean were removed from the analysis. Table 3 displays the goodness scores granted to the solution alternatives as a function of condition. An ANCOVA analysis with condition as a between-subjects factor and time on task as a covariate confirmed a significant difference between conditions on their preference scores for the shift-alternative, *F*(2, 253) = 4.01, *p* = .02, *η^2^_p_* = .03. As predicted, a planned comparison revealed that participants in the shift-priming condition assigned higher scores to the shift solution than participants in the mirror-priming condition *t*(166.9) = 1.99, *p* < .05, Cohen’s d = .31, 95% CI = [.12, 25.8]. As opposed to the procedure of Experiments 1 and 2, where the constraint to make the scores of all alternatives add to 100 rendered scores to the mirror solution largely dependent on the scores to the shift solution, in the present experiment the possibility of granting scores from 1 to 100 allowed participants to assess the mirror solution quite independently from the shift alternative. An ANCOVA analysis with condition as a between-subjects factor and time on task as a covariate confirmed a significant difference between conditions on their preference scores for the mirror-alternative, *F*(2, 253) = 8.11, *p* < .001, *η^2^_p_* = .06. As predicted, a planned comparison revealed that participants in the mirror-priming condition assigned higher scores to the mirror solution than participants in the shift-priming condition, *t*(153.29) = 3.11, *p* < .01, Cohen’s d = .48, 95% CI = [6.63, 29.61]. These results confirm that the visuospatial schemas thought to underlie meta-analogical transfer can influence later analogizing in and by themselves. As these dynamic schemas were not incorporated into a preparatory episode of analogical reasoning, a standard account of our results based on the transfer of predicate mappings does not hold.

**Table 3:** Preference scores as a function of priming condition and type of solution, Experiment 3.


TASK AND PRIMING CONDITION	N	TIME	SHIFT-SCORES		MIRROR-SCORES		DISTRACTOR SCORES	
		
			MEAN	SD	MEAN	SD	MEAN	SD

Ambiguous analogy task								

control	87	72.1	40.59	41.73	82.64	30.95	1.22	1.36

mirror	85	87	45.13	40.72	80.73	31.5	117	1.02

shift	85	76.6	58.09	44.01	62.61	43.4	1.29	2.11

Ambiguous analogy task (unaware participants only)								

control	77	73	40.99	41,47	83.58	29.81	1.13	1.03

mirror	61	89.8	51.49	40,19	80.02	30.54	1.21	1.2

shift	58	76.2	62.71	43,03	62.07	42.42	1.36	2.5

Hinted-transfer task								

control	87	56.1	44.7	43.4	67.3	39.9	10.4	24.8

mirror	85	54.2	28.7	40.2	77.8	38.9	5.3	17.3

shift	85	54.7	76.7	41.3	31.6	43	4.47	15.8


When assessing priming effects on cognitive tasks as complex as our letter-string analogies, an interesting dimension of inquiry concerns the extent to which the observed effects are contingent on participants being consciously aware of a relation between the priming stimulus and the target task. An analysis of participants responses to our post-task awareness questionnaire revealed that roughly one third (32.3%) of participants in the primed conditions spontaneously perceived a relation between the priming stimulus and the ambiguous letter-string analogy. Albeit exploratory, a one-way ANOVA on the subset of participants who failed to perceive an association between these two phases revealed significant differences across priming conditions on the goodness scores assigned to the shift-based solution, *F*(2, 193) = 4.54, *p* = .012, *η^2^_p_* = .04. Post hoc analyses using a Holms correction for family-wise comparisons revealed that participants receiving a shift visuospatial schema granted higher goodness scores to the shift-based solution than participants receiving an unrelated visuospatial schema, *M* = 62.51, *SD* = 43.03 vs. *M* = 40.99, *SD* = 41.47, *p* < .01, all other *p*s > .05. Likewise, a one way ANOVA on participants scores to the mirror solution revealed significant differences across priming conditions, *F*(2, 193) = 7.11, *p* = .001, *η^2^_p_* = .07. Post hoc analyses using a Holms correction revealed that participants receiving either a mirror visuospatial schema (*M* = 80, *SD* = 30.5) or an unrelated visuospatial schema (*M* = 83.6, *SD* = 29.8) granted higher goodness scores to the mirror solution than participants receiving a shift visuospatial schema (*M* = 62.1, *SD* = 42.4), *p*s = .009 and .001 respectively. Hence, the present results show evidence that the influence of dynamic visuospatial schemas on solutions to an analogy problem can take place even when participants do not relate the schemas to the problem. This finding is relevant to discounting possible competing interpretations of the main results of Experiment 3: that participants might have formed descriptive predicates describing the animation, and then transferred the predicates to the target analogy. It should be noted, however, that such an explicit and conscious verbalization/description would make it far more likely that they would judge the animation as playing a role in their ratings of the ambiguous analogy, and the present data argue against that for two-thirds of participants. Since part of perceptual-motor processing is deemed unavailable to conscious thought, the data cohere with the interpretation that for many participants, pre-symbolic perceptual-motor processes were primed when watching the animations, and had a carry-over effect on the analogy ratings of Experiment 3.

Even though the goodness scores assigned to the shift alternative were statistically higher among participants receiving a shift priming (62/100) than among participants receiving an unrelated priming (41/100), the fact that they seem to be far from 100/100 would intuitively give the impression of a relatively weak effect. To gain a psychologically more realistic estimate of ceiling performance than the maximum theoretical goodness score of 100/100, the last segment of the procedure re-exposed participants to the previously seen animation, and explicitly asked them to reassign goodness scores to the previous solutions in a manner consistent with the animations. The goodness scores granted to the shift solution under this most directive indication were of 76.7/100 among shift-primed participants, vs. 44.7/100 among participants receiving an unrelated priming (for a parallel analysis of participants scores to the mirror solution, see Table 3). Considering this more realistic estimate of ceiling performance, the effect of the priming manipulation looks rather intense, even among participants who were unaware of a relation between the priming stimulus and the subsequent problem.

## General Discussion

A growing set of studies (see e.g., Goldwater & Jamrozik, 2019; Simms & Richland, 2019 or Vendetti et al., 2014) has demonstrated that a broadly construed mindset to look for relational structures can be transferred across episodes of analogical reasoning. Along with previous results of Burns (1996) and Pulido (2002), the results of our first two experiments reach beyond the mindset studies in demonstrating that specific features of the analogies themselves can also be transferred across instances of analogical reasoning. In a third experiment, a similar transfer effect was elicited by the previous presentation of a brief visuospatial animation, thus suggesting that the carry-over of perceptual schemas likely mediated the transfer effects obtained in Experiments 1 and 2.

Upon documenting order effects during the solution of letter-string analogies, Burns (1996) postulated that the medium through which meta-analogical transfer operates is the carry-over of predicate mappings across episodes of analogical reasoning. While this interpretation represented a reasonable account of his results, it cannot explain the fact that only mappings between similar concepts proved to transfer reliably. Mappings between dissimilar concepts, as in Hofstadter’s “first lady” analogy, often fail to be transferred (Pulido, 2002, see Pulido et al. 2010 for a review). As the “transfer of predicate mappings” hypothesis fails to discriminate between these two relevant cases, it seems insufficient to comprehensively account for the available data.

Based on a growing number of studies showing that dynamic visuospatial schemas can inadvertently bias participants’ responses to a wide range of problem-solving situations (e.g., Grant & Spivey, 2003; Goldstone et al, 2010; Livins et al., 2016; Pedone et al., 2001), we advanced the idea that the activation and carry-over of visuospatial schemas could potentially explain the observed patterns of transfer between letter-string analogies. For the ambiguous analogies used in the present study, a rotation about the vertical axis represented a more frequent way of interpreting the transformation between the A and the B terms than the alternative “shift” operation. Hence, the fact that a priming analogy designed to elicit a shift operation favored a shift-based solution to a later analogy suggests that such visuospatial operation remained available for perceptual and/or conceptual processing, thus promoting an interpretation of the A®B transformation that went against some natural resistance, and which was not elicited by default. To gather stronger evidence for the processes presumed to underlie the facilitation effects observed in Experiments 1 and 2, in Experiment 3 a similar effect was elicited by presenting the postulated visuo-spatial schemas directly, that is, without being embedded within a preparatory analogy. This result strongly suggests that visuo-spatial processes can be carried over to a subsequent analogy, and this in turn makes it more plausible that the carry-over of visuo-spatial schemas was involved in Experiments 1 and 2. Without denying that predicate mappings could conceivably be transferred across analogy episodes under certain conditions, the results of our experiments provide a more complete account of meta-analogical transfer than was previously available, one that can more comprehensively explain the inconsistent effects of meta-analogical transfer observed in the literature.

A rather unexpected finding of Experiment 3 concerns the high proportion of participants who did not perceive a connection between the priming animations and the test problem. The fact that similar group differences were obtained with this subset of participants discards the possibility that the main effects observed in Experiment 3 with the whole sample were driven by a minority of participants who explicitly related the test analogy to the previous animation. Even though earlier studies had demonstrated that both animations and induced attentional patterns (e.g., saccades) were capable of biasing participants responses to reasoning tasks, evidence that most participants can be biased without explicitly perceiving a connection came mostly from studies whose priming phase induced oculomotor displacements either explicitly (Thomas & Lleras, 2007) or implicitly (Livins et al., 2016). The present results provide evidence that priming effects can remain unnoticed even when the representational nature of the prime and the test item is more conspicuous, thus suggesting that the effects obtained in studies like Pedone et al. (2001) or Goldstone et al. (2010) might likewise have escaped conscious awareness. More broadly, the present study resonates with a growing set of studies highlighting the role of perceptually-represented structure in the transfer processes that take place during problem-solving (Catrambone et al., 2006; Kubricht et al., 2017) and explanatory model construction (e.g., Clement, 2022; Lakoff & Nuñez, 2013). These studies suggest that current theoretical and computational models of analogical reasoning might be improved by including perceptual information as part of their representational schemas (Pedone et al., 2001).

The present results bear important implications for the use of analogies during standardized testing. Many test batteries widely used for assessing intellectual abilities often include a series of analogies. In these cases, the fact that solutions to a first analogy might help or hinder solutions to subsequent analogy episodes can potentially exert an uncontrolled effect on performance, negatively affecting the consistency of the tests. This danger seems especially challenging for *adaptive testing* schedules, where performance on an initial set of items determines the selection of the items to be subsequently presented. In light of the results from Experiment 3, where participants’ appraisal for alternative solutions was primed by a stimulus that did not take part in an analogical episode, it is likely that particular aspects of a problem’s representation might affect performance of subsequent problems in a series, even if they are neither analogous to the previous problems nor analogical in nature.

The priming effects obtained in the present study can also have implications for instruction. In contrast to incidental stimuli such as a Goldstone et al.’s “background grating”—which will only serendipitously match the perceptual structure of an intended operation—, the instructional schemas that are usually presented in support of verbal descriptions tend to be consistent with an intended solution. The case of teachers’ gesturing is especially interesting in this context, given that they are often generated as cognitive aids for the speaker’s own reasoning, and hence fall outside of their conscious control (McNeill, 1992). When the teachers’ gestures are still compatible with the target situation as viewed from the students’ spatial perspective (e.g., an upward motion of the arms or a symmetric horizontal displacement), our results would suggest that participants will inadvertently capitalize on them in the service of subsequent processing related to such gesturing. However, this advantage is likely to reverse in those cases where the gesture is appropriate from the instructor’s frame of reference, but no longer matches the situation as perceived by the students (e.g., a left-to-right displacement or a clockwise motion). Further studies should address how this priming phenomenon might affect instructional practices, as well as how it can eventually be leveraged to aid students’ learning and performance.

## Data Accessibility Statement

Raw data from all three experiments can be downloaded at osf.io.

## Footnotes

Note that the triplet representing the “C” term in the analogy involves a reverse alphabetical order.
